# Down-regulation of estrogen-related receptor alpha (ERRα) inhibits gastric cancer cell migration and invasion *in vitro* and *in vivo*

**DOI:** 10.18632/aging.202508

**Published:** 2021-02-11

**Authors:** Yuejiao Zhong, Kang He, Lin Shi, Lingxiang Chen, Bin Zhou, Rong Ma, Hui Yu, Jia Zhang, You Shuai, Yan Fei, Jianwei Lu

**Affiliations:** 1 Department of Medical Oncology, Jiangsu Cancer Hospital and Jiangsu Institute of Cancer Research and The Affiliated Cancer Hospital of Nanjing Medical University, Nanjing 210009, Jiangsu Province, China; 2 Department of General Surgery, Jiangsu Cancer Hospital and Jiangsu Institute of Cancer Research and The Affiliated Cancer Hospital of Nanjing Medical University, Nanjing 210009, Jiangsu Province, China; 3 Department of Central Lab, Jiangsu Cancer Hospital and Jiangsu Institute of Cancer Research and The Affiliated Cancer Hospital of Nanjing Medical University, Nanjing 210009, Jiangsu Province, China; 4 Department of Invasive Technology, Jiangsu Cancer Hospital and Jiangsu Institute of Cancer Research and The Affiliated Cancer Hospital of Nanjing Medical University, Nanjing 210009, Jiangsu Province, China; 5 Department of Imaging, Jiangsu Cancer Hospital and Jiangsu Institute of Cancer Research and The Affiliated Cancer Hospital of Nanjing Medical University, Nanjing 210009, Jiangsu Province, China; 6 Department of Oncology, The Affiliated Suqian First People's Hospital of Nanjing Medical University, Suqian 223800, Jiangsu Province, China

**Keywords:** estrogen-related receptor alpha, gastric cancer, epithelial–mesenchymal transition

## Abstract

Objective: To investigate the correlation between estrogen-related receptor a (ERRα) expression level and gastric cancer (GC).

Methods: We collected GC and adjacent normal tissues from 50 patients. The parameters of the patients were summarized, and correlation with the expression level of ERRα was calculated. Downregulated ERRα using lentivirus was designed and transfected to SGC-7901 and MGC-803 cells. Cell migration, invasion and wound assays were conducted to determine the correlation between ERRα and capacity for cell migration and invasion. The expression level of the genes involved in epithelial–mesenchymal transition, including E-cadherin, γ-catenin, N-cadherin and vimentin, was determined via real-time or quantitative polymerase chain reaction(qPCR) and Western blot analysis.

Results: The expression of ERRα tends to be higher in GC tissues than in adjacent normal tissues. Analyses ofthe expression level of ERRα and patient parameters show that the ERRα level is significantly correlated with TNM staging and patient survival (*P*<0.05). The downregulation of ERRα can inhibit cell invasion and migration, which was proven by Transwell and cell wound assays. The levels of E-cadherin and γ-catenin increased by conducting qPCR and Western blot analysis. Meanwhile, the levels of N-cadherin and vimentin decreased when ERRα expression was reduced.

Conclusion: ERRα is highly expressed in GC tissues and can promote the migration and invasion of cancer cells. It can be a potential marker for GC diagnosis.

## INTRODUCTION

Gastric cancer (GC) is the fourth most common malignancy worldwide and the second cause of cancer-related deaths in the world [1, 2]. GC results from a combination of environmental and specific genetic factors. In European countries, the five-year survival rate varies from 10% to 30% [3]. Carbohydrate antigen 19-9 and carcinoembryonic antigen are common biomarkers for GC diagnosis, but their specificity and sensitivity are insufficiently high [4]. Therefore, novel biomarkers should be identified for GC diagnosis.

Estrogen-related receptor alpha (ERRα), an orphan nuclear receptor, is important in regulating the signaling pathway in cancer. The activities of ERRα and its co-regulators, namely, PGC-1a and PGC-1b, depend on the expression level of ERRα [5]. ERRα can regulate various enzymes involved in glycolysis, lipid and amino acid metabolism and tricarboxylic acid cycle, and thus, accommodate energy to cancerous cells [5–8]. A specific pattern observed between estrogen receptor alpha (ERRα) and ERRα is that the former binds to estrogen response elements (EREs), whilst the latter binds to a distinct genomic motif called ERRE [6]. ERα directs gene expression in cell development and proliferation, and ERRα target genes exhibit metabolic-related functions associated with breast cancer [6]. Accumulating evidence showed that ERRα is involved in tumorigenesis and cancer development in prostate cancer, oral squamous cell cancer, colon cancer and gallbladder cancer [9–12]. It can regulate the transcription of genes, such as CCNE1, OPN, WNT11 and OPG, all of which are important effectors in proliferation, metabolism and metastasis in cancer progression [7]. Bioenergetics in tumor tissues facilitates the growth of tumor, which involves ERRα as an important adaptive regulator [13]. Besides, ERRα positivity is associated with severer invasion and higher risk of relapse [14–16]. Given that ERRα plays a role in multiple cancers via various mechanisms, and the correlation between ERRα and GC has not been reported, it is of significant value to explore the role of ERRα in gastric cancer.

In the present work, we collected specimens from patients with GC and determined the expression of ERRα via tissue array. We also designed an ERRα low-expression vector and downregulated the expression of ERRα in SGC-7901 and MGC-803 cell lines to verify the functions of ERRα.

## RESULTS

### Expression levels of ERRα in GC

**Figure 1:**
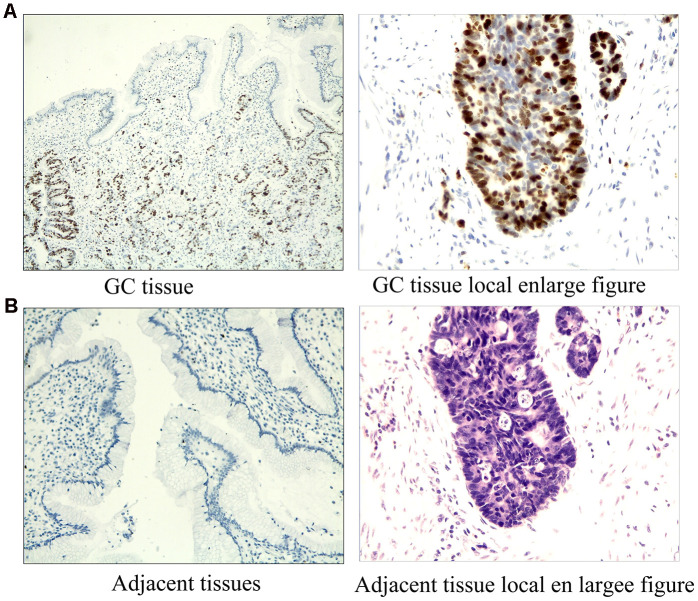
**Immunostaining results of ERRα in GC tissues and adjacent tissues.** (**A**) Immunostaining results of ERRa in GC tissues; (**B**) Immunostaining results of ERRa in adjacent tissues.

**Figure 2:**
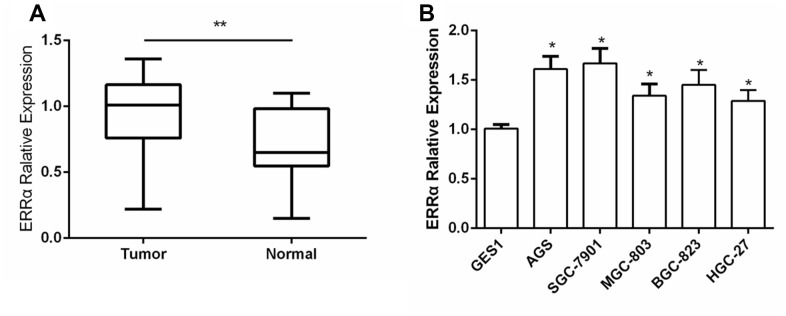
**The expression of ERRα in GC tissues and GC cell lines.** (**A**) The expression of ERRa in GC tissues; (**B**) The expression of ERRa in GC cell lines *P<0.05, **P<0.01.

The expression of ERRα was determined via tissue array. We collected 50 cases of GC and adjacent tissues for detection. Immunohistochemical results showed that the expression of ERRα in GC tissues was higher than that in adjacent tissues (Figure 1A, 1B). The mRNA expression level of ERRα was 0.97±0.27 in GC tissues and 0.74±0.25 in adjacent tissues (Figure 2A). Analysis showed that the expression level of ERRα in GC tissues was significantly higher than that in adjacent tissues (*P*<0.01). A high expression of ERRα was also identified in the five GC cell lines (AGS, SGC-7901, MGC-803, BGC-823 andHGC-27) compared with that in GES1 (*P*<0.05, Figure 2B). We categorised tissues as ERRα high or low expression the basis of the median. Amongst the GC tissues, 26 had high ERRα expression and 24 had low expression.

### Clinical value of ERRα expression for patients with GC

**Figure 3:**
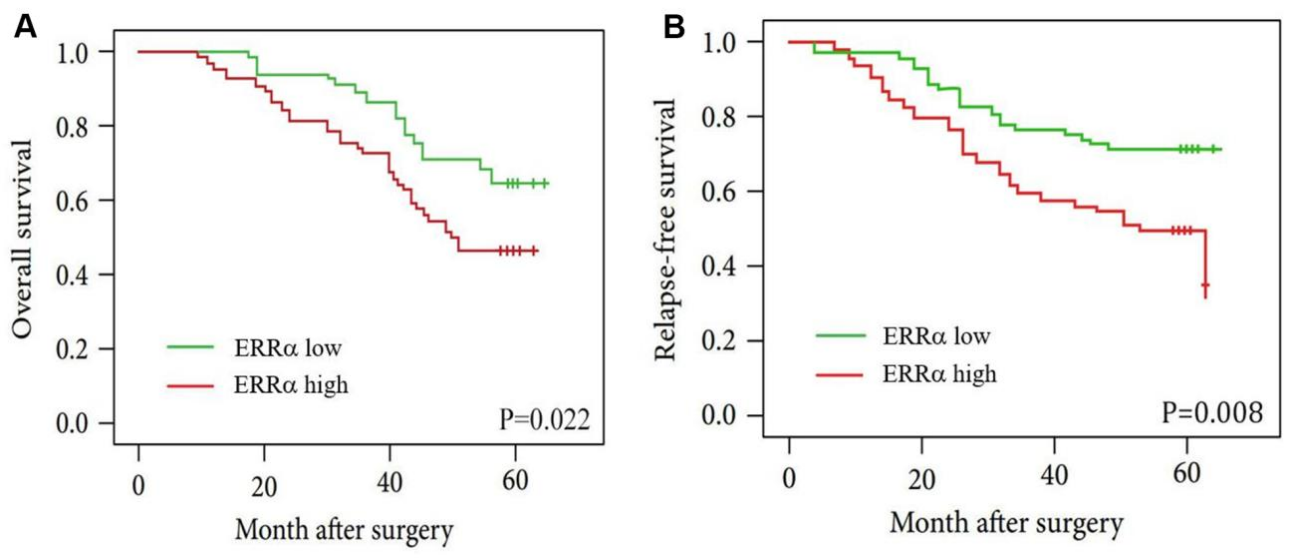
**Kaplan-Meier curves for patients with low ERRα expression versus high ERRα expression.** (**A**) OS curves of patients with low and high expression of ERRα, P=0.022; (**B**) RFS curves of patients with low and high expression of ERRα, P=0.008.

To study the clinical significance of ERRα in GC, we analysed the clinical relationship between the expression of ERRα in patients with GC and clinicopathological characteristics. The results showed that a high expression of ERRα was associated with advanced TNM stage (Table 1, *P*=0.002). Furthermore, the Kaplan–Meier analysis indicated that patients with high ERRα expression exhibited a decrease in OS (Figure 3A, *P*=0.022) and relapse-free survival (Figure 3B, *P*=0.022). The multivariate analysis results showed that a high expression of ERRα was a significant independent prognostic factor for OS in patients with GC (Table 2, *P*=0.024).

**Table 1:** Relationship between ERRα expression and clinicopathologic features of GC patients.

**Characteristic**		**ERRα expression**		**p-value**
		**Low(n=24)**	**High(n=26)**	
Sex				0.612
Male		15	17	
Female		9	9	
Age(yr)				0.504
<50		7	8	
≥50		17	18	
Differentiation				0.356
Good/Moderate		15	18	
Poor		9	8	
Depth of invasion				0.115
mucosa		10	12	
Submucosa		14	14	
TNM staging				0.002*
I~II		7	16	
III~IV		17	10	
Lymph node metastasis				0.324
Yes		13	17	
No		11	9	

p-value was acquired by Pearson chi-square test.

*Statistically significant (p<0.05).

**Table 2:** Multivariate analysis of several variables for RFS and OS.

**Variable**	**Univariate Cox’s**		**Multivariate Cox’s**	
	**Regression analysis**		**Regression analysis**	
	**Hazard ratio**	**p-value**	**Hazard ratio**	**p-value**
	**(95% CI)**		**(95% CI)**	
ERRα (high vs. low)	2.733	0.005	1.621	0.024*
	(1.341-4.992)		(1.033-2.152)	
Sex (male vs. female)	1.108	0.798	-	-
	(0.741-1.439)			
Age (<50 yr vs. ≥50 yr)	0.772	0.346	-	-
	(0.468-1.323)			
Depth of invasion	1.177	0.228	1.489 (0.733-3.308)	0.025*
	(0.941-1.959)			
TNM staging (I~II vs. III~IV)	2.108	0.002	2.314 (1.078-3.216)	0.032*
	(0.985-3.424)			
Differentiation (poor vs.good/moderate)	1.338	0.539	-	-
	(1.044-2.118)			
Lymph node metastasis (Yes vs. No)	1.446	0.013	1.518 (1.031-2.164)	0.010*
	(1.023-2.048)			

p-value was acquired by Cox proportional hazards regression.

*Statistically significant (p<0.05).

RFS, recurrence-free survival; OS, overall survival; CI, confidence interval.

### Downregulation of ERRα inhibited proliferation, invasion and migration of GC Cells

**Figure 4:**
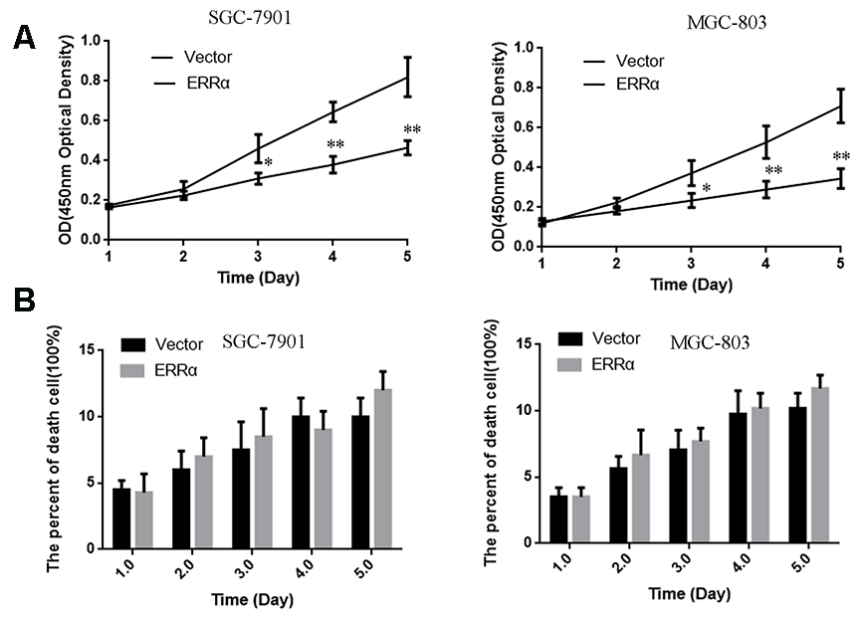
The cell proliferation and death were measured by CCK-8 (**A**) and Trypan Blue exclusion assays (**B**). (**A**) The cell viability were measured using CCK-8 assay. (**B**) The death cell population was analyzed using Trypan Blue dye exclusion assay. Data are presented as the mean ± SD. **p < 0.01, *p < 0.05 vs. Vector group.

**Figure 5:**
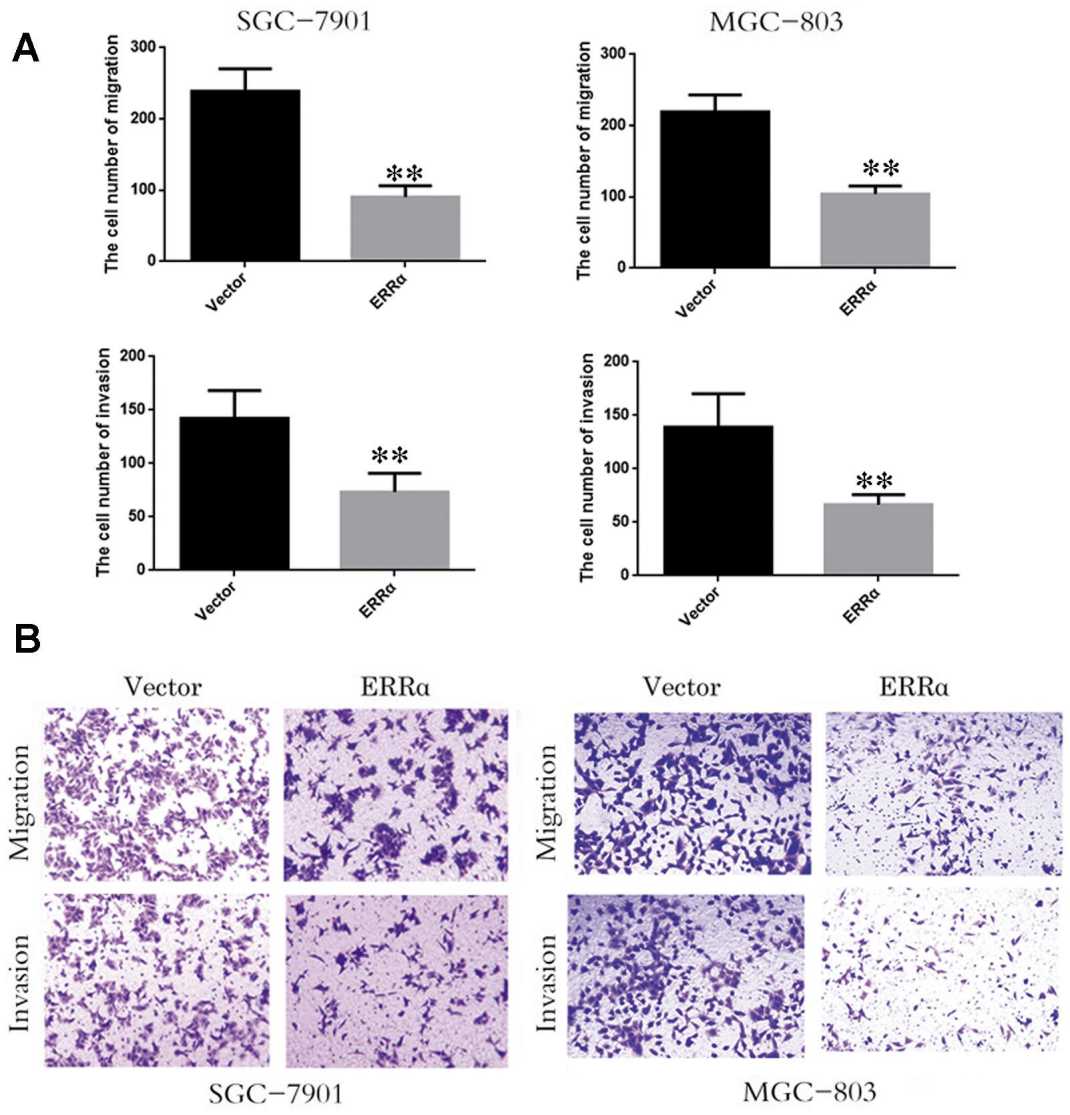
**Low expression of ERRα inhibits the cell migration and invasion.** (**A**) The cell number of migration and invasion in SGC-7901 cell and MGC-803. (**B**) The migration and invasion ability of SGC-78901 and MGC-803 cells were observed by crystal violet staining **P<0.01.

SGC-7901 and MGC-803 cell lines were used to perform cell proliferation, migration and invasion assays. The ERRα vector was down regulated for transfection to downregulate the ERRα level in cells. An empty vector was used as control. Western blot analysis was conducted to determine the ERRα level in cells. The results showed that the low expression of the ERRα vector evidently decreased the expression of ERRα in SGC-7901 and MGC-803 cells compared with the empty vector (data not show). Therefore, the vector was successfully transfected into the cells. The trypan blue rejection method and CCK-8 assay were used to measure the cell proliferation and viability. The results showed that the knocking down ERRα can significantly inhibit proliferation capabilities without affecting the cell death (Figure 4). The cells transfected with the vectors were then used for cell Transwell migration and invasion assays. Cells that migrated to or invaded the bottom chamber were stained and counted. The results indicated that the downregulation of ERRαsignificantly attenuated the capacity for cell migration and invasion in SGC-7901 and MGC-803 cells (Figure 5A, 5B).

### Wound scratch assay

**Figure 6:**
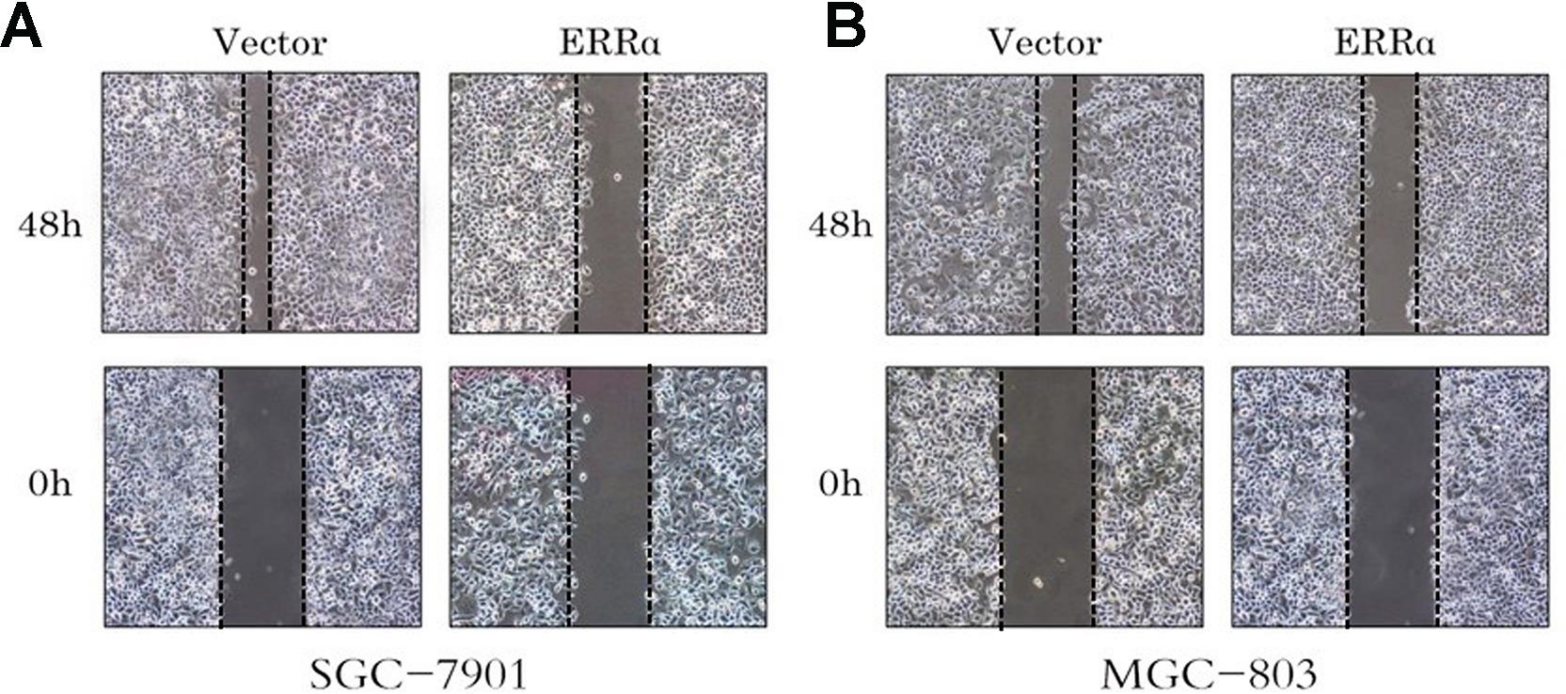
**Cell wound assay indicates the cell migration of the SGC-7901 and MGC-803 cells were inhibited by the low expressed ERRα.** (**A**) Cell wound assay indicates the cell migration of the SGC-7901 was inhibited by the low expressed ERRa; (**B**) Cell wound assay indicates the cell migration of the MGC-803 was inhibited by the low expressed ERRa.

We also performed cell wound scratch assay. The wound was recorded at 0, 24 and 48h after it was generated. The downregulation of ERRα can weaken cell migration significantly in SGC-7901 and MGC-803 cells, particularly MGC-803 cells (Figure 6A, 6B).

### Target gene of ERRα

**Figure 7:**
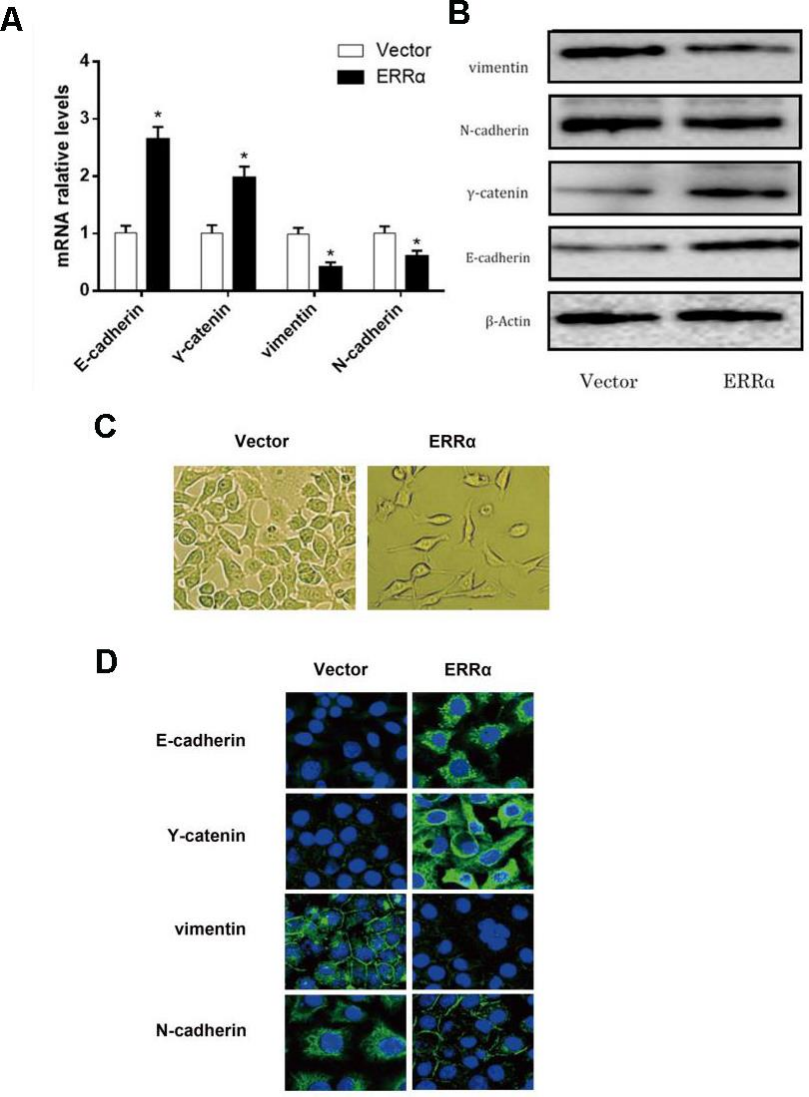
**mRNA level and protein level of the EMT pathway related genes determined by qRT-PCR, western blot and fluorescence staining.** (**A**) mRNA expression level of E-cadherin, γ-catenin, N-cadherin and vimentin; (**B**) Protein expression level of E-cadherin, γ-catenin, N-cadherin and vimentin. (**C**) morphology of cells undergoing EMT; (**D**) Fluorescence staining of E-cadherin, Y-catenin, vimentin, and N-cadherin. *P<0.05.

To verify the mechanism of ERRα in regulating cell migration and invasion, qPCR and Western blot analysis were conducted to determine the mRNA and protein levels of E-cadherin, γ-catenin, N-cadherin and vimentin, which are involved in the epithelial–mesenchymal transition (EMT) signaling pathway. The results showed that the mRNA and protein levels of E-cadherin and γ-catenin were increased, while the levels of vimentin and N-cadherin decreased in both cell lines (Figure 7A, 7B), indicating that the EMT signaling pathway was inhibited and potentially improved cell migration and invasion when ERRα had a low expression. To further illustrate the EMT stage cells, we performed immunofluorescence staining to more closely observe the cell morphology and differentiation occurred during EMT. As shown in Figure 7C, the cell density of cells with ERRα downregulated is lower than control. The immunofluorescence staining of cells presents consistent result with western blot, and the cells in control group looks less edged.

### Downregulation of ERRα inhibited the growth of GC cells *in vivo*

**Figure 8:**
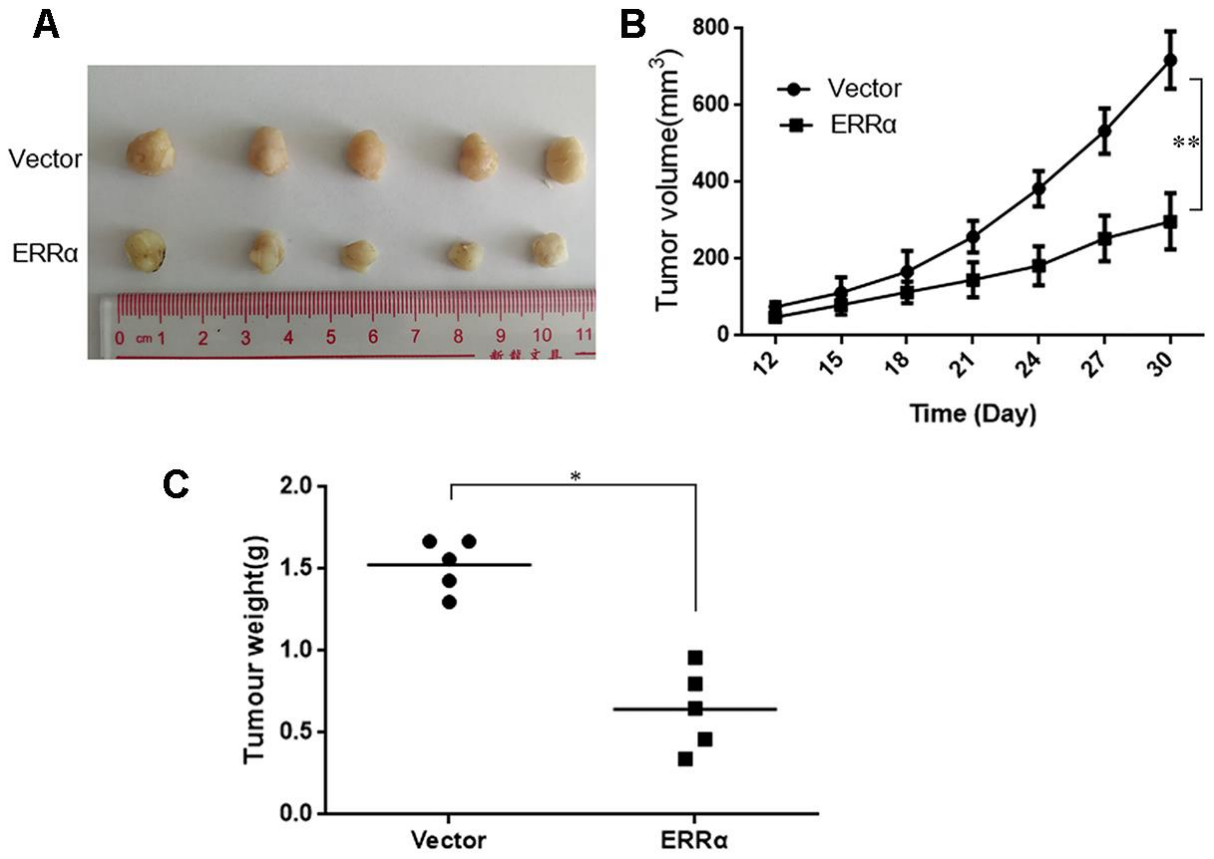
**Knockdown of ERRα inhibits the proliferation of gastric cancer cells *in vivo*.** MGC-803 cells (ERRα and Vector) were subcutaneously inoculated into BALB/c nude mice and tumor growth was monitored. 10 days after tumor inoculation, tumor volume was measured every three days. On the 30th day, mice were sacrificed and tumor xenografts were excised from surrounding tissue and weighted. (**A**) Representative xenograft tumors for indicated cells were shown. (**B**) ERRα knockdown significantly reduced xenograft tumor growth in male nude mice by tumor volume examination. (**C**) ERRα knockdown significantly suppressed xenograft tumor weights. Data represent mean ± S.D, *P < 0.05, **P < 0.05.

To assess the antitumor activity of ERRα *in vivo*, MGC-803 cells (shERRα and vector) were subcutaneously inoculated into BALB/c nude mice and tumor growth was monitored. As shown in Figure 8A, 8B, the knockdown of ERRα can significantly inhibit tumor growth compared with the vector. The knockdown of ERRα significantly decreased tumor volume and weight (Figure 8B, 8C, *P*<0.05; Figure 5B, *P*<0.05). Overall, these findings indicated that knocking down ERRα can suppress GC cell growth *in vivo*.

## DISCUSSION

In this study, we found that the expression level of ERRα tends to be higher in GC tissues. The expression level of ERRα is significantly correlated with TNM staging and the survival rate of patients. In general, a high expression of ERRα is correlated with poor prognosis in patients with GC. The results of the assays performed n SGC-7901 and MGC-803 cells proved that the low expression of ERRα suppressed the capacity for cell migration and invasion and regulates the EMT signaling pathway.

ERRα is also highly expressed in breast and ovarian cancers and may predict poor prognosis [8, 17, 18]. The downregulation of ERRα activity by pharmacological antagonists decreased cell proliferation and tumorigenicity in ER-positive and ER-negative breast cancer [19–21]. Therefore, our results presented a consistent outcome compared with evidence in breast and ovarian cancers. We evaluated the expression level of ERRα in GC for the first time. We suspected that the specific feature of ERRα expressed in GC. Interestingly, Subrata et al. showed that the expression level of ERRα is higher in basal-like and ER-negative tumors than in luminal and ER-positive tumors [22]. On the basis of evidence, we hypothesized that the expression level of ERRα may not be an independent factor that regulates GC processes. The functions of ERRα can be complicated and it may require combining with ERα in performing its functions.

The results of this study indicated that cells with low ERRα expression present weaker capacity for cell migration and invasion, which we suspected has been inhibited by the EMT signaling pathway. The EMT process has not gained considerable attention for its potential role in converting benign tumors into invasive and metastatic tumors [23–26]. During the EMT process, the expression of junction proteins and other epithelial characteristic functions are lost. Eventually, tumor cells lose their capacity to interact with one another and gain increased migratory capacity due to increased expression of mesenchymal proteins, such as vimentin and a-SMA [27]. Therefore, tumor cells acquire the capacity to migrate during the EMT process, invade surrounding tissues and subsequently spread via blood and lymphatic vessels [27]. E-cadherin is the major component that contributes to epithelial cell-to-cell interaction [28]. Ahigh expression of E-cadherin is typically detected during carcinoma progression and is commonly considered a marker for tumor cell invasion [29]. With the exception of E-cadherin, the expression of mesenchymal genes, such as Snail, vimentin and fibronectin, is upregulated [27]. Moreover, other markers, such as N-cadherin and γ-catenin, can be used to assist in the diagnosis of tumor cell invasion. Our results showed that the expression levels of E-cadherin and γ-catenin were increased, but the levels of vimentin and N-cadherin were decreased. Therefore, it can help improve adherent junctions between cells and prevent cell invasion. Eventually, tumor cell migration and invasion can be inhibited by the downregulation of ERRα. The results indicated that ERRα can be a pro-cancer factor. It can promote the migration and invasion of tumor cells, worsening the prognosis of patients with GC.

In this study, we demonstrated that ERRα can be a potential marker for GC. Its expression increases in patients with GC, leading to worse prognosis. ERRα can play the role of tumor promoter, and it can promote cell migration and invasion by increasing the levels of E-cadherin and γ-catenin and decreasing the levels of N-cadherin and vimentin. In future research, the number of participating patients can be increased to provide more persuasive evidence between ERRα and GC. The regulatory network of ERRα in GC can also be investigated to present a comprehensive understanding of the functions of ERRα.

## MATERIALS AND METHODS

### Collection of tissue specimens

GC and adjacent tissues were collected from 50 patients diagnosed in Jiangsu Cancer Hospital from 2017 to 2019. The samples were immediately fixed in 10% formalin solution for 14 h. Then, paraffin embedment was performed for further immunohistochemical study. Pathological stage was determined on the basis of Fuhrman nuclear grade. Tumor stage was determined in accordance with the TNM classification of the American Joint Committee on Cancer. Prognosis assay was performed to follow-up with survivors and estimate the overall survival (OS) rate. OS was calculated from the date of surgery to the date of death or the latest follow-up. The present study was approved by the Ethics Committee of Jiangsu Cancer Hospital. All the participants provided a signed informed written consent in advance.

### Tissue array and immunohistochemistry staining

Human GC tissue microarray was constructed using formalin-fixed and paraffin-embedded kidney tissues. Immunohistochemical studies were performed using a Leica autostainer XL ST5010 (Leica Biosystems, Wetzlar, Germany). The sections were dehydrated with ethanol and then subjected to antigen retrieval in citrate buffer (10 mM, pH 6.0) for 30 min. The sections were blocked in peroxidase-blocking reagent (Dako Cytomation, Glostrup, Denmark) for 15 min at room temperature, followed by incubation with rabbit anti-human polyclonal primary antibody (1:1000 dilution; Abcam, Cambridge, UK) overnight at 4° C in a humidified chamber. Sections were then incubated with mouse anti-rabbit secondary antibody (1:1500 dilution; Abcam, Cambridge, UK) for 30 min at RT. After washing completely with phosphate-buffered saline (PBS), the sections were developed in freshly prepared diaminobenzidine solution, counterstained with haematoxylin, dehydrated through graded ethanol, cleared with xylene and cover-slipped. The expression of ERRa was estimated in accordance with the percentage of stained cells. Each sample was classified either low-expression (≤30%) or high-expression (>30%).

### Cell culture

GC cells (AGS, SGC-7901, MGC-803, BGC-823and HGC-27) and human normal gastric epithelial cell (GES1) were obtained from the Shanghai Cell Bank (Shanghai, China) and cultured at 33° C with 10% CO_2_. Dulbecco’s modified Eagle’s medium (DMEM) in high glucose with 10% fetal bovine serum (FBS; Gibco, USA) was supplemented. Cells were cultured, amplified and passaged. After 3 days, the cells were digested and pelleted via centrifugation. Cell morphology was observed using a light microscope, and the cells were suspended to a concentration of 1×10^6^/mL.

### Cell transfection

The lentiviral shRNA expression vector targeting shERRα and scrambled control were obtained from Obio Technology (Shanghai, China). Cells were seeded into six-well plates at a density of 2×10^5^ cells/well. After 80% confluence was reached, shERRα (ERRα)and scrambled control siRNA (vector) were transfected into cells. After 48 h, real-time or quantitative polymerase chain reaction(qPCR) and Western blot analysis were performed to determine transfection efficiency.

### qPCR

The mRNA level of ERRα was verified via qPCR. M-MLV reverse transcriptase (Promega, USA) was used to synthesise cDNA. PCR was prepared with GoTaq qPCR Master Mix (Promega, USA) and performed on an ABI 7500 system (Applied Biosystem, USA). GAPDH was used as housekeeping gene to perform comparative quantification.

### Western blot analysis

Cells were lysed in 1% sodium dodecyl sulphate (SDS) lysis buffer. Bicinchoninic acid (BCA) assay was performed to determine protein concentration. SDS-polyacrylamide gel electrophoresis (10%) was used to separate the protein. The protein was then transferred to a nitrocellulose membrane. Nonfat milk in PBS was used to block the membrane at room temperature for 1h. The membrane was incubated overnight at 4° C with primary antibody (Abcam, UK). After washing several times with PBS, the membrane was incubated in blocking buffer with a secondary antibody coupled to horseradish peroxidase for 2h at room temperature. Complexes were formed on the membrane, and the membrane was detected using ECL Plus (Amersham Biosciences/GE Healthcare, Velizy, France).

### Migration and invasion assays

A 24-well plate containing 8mmpore size chamber inserts (Corning, USA) was used to evaluate the migration and invasion of tumor cells. For the migration assay, 1×10^5^ cells were seeded into the upper chamber. For the invasion assay, the membrane was coated with Matrigel (BD Biosciences, USA) to form a matrix barrier, and then 2×10^5^ cells were placed in the upper chamber. In each lower chamber, 600mLof DMEM with 10% FBS was added. Cells were incubated at 37° C and allowed to migrate for 36h or invade for 48h. After incubation, the cells that migrated through the pores were fixed with 4% paraformaldehyde and stained with 0.1% crystal violet. Then, the cells were counted and photographed under an IX71 inverted microscope (Olympus, Tokyo, Japan).

### Wound scratch assay

The cells were seeded into 12-well plates, cultured to confluence and wounded by scraping with a 0.1–10 μL pipette tip. The cells were washed with PBS. The cell culture was then re-fed with the medium for 24h or 48h. The cells were then fixed with formaldehyde and stained with 0.1% toluidine blue at room temperature for 30min. Images of the cells were captured using a camera-equipped microscope (Carl Zeiss, USA).

### *In vivo* tumorigenesis assay

Male BALB/c nu/nu mice (4–5 weeks old) purchased from the Laboratory Animal Centre of Shanghai, Chinese Academy of Sciences (Shanghai, China), were housed under specific pathogen-free conditions. The mice were randomly divided into two groups (5 mice/ group). MGC-803 cells (shERRα and vector) were subcutaneously inoculated into the BALB/c nude mice, and tumor growth was monitored. Viable cells (3×10^6^ cells/group) were injected subcutaneously into the flanks of the mice. Then, 10 days after cell injection, the length (*L*) and width (*W*) of tumor xenografts were measured with a Vernier calliper at four- or three-day intervals. Tumor volumes were calculated (*V* = *W*^2^ × *L*/2). The animals were sacrificed under general anaesthesia with chloral hydrate (5%, 100 μL/10 g).

### Statistical analysis

The association between ERRα expression and pathological parameters was calculated via the Chi-squared test. ERRα expression between normal and GC samples was compared using Student’s two-tailed *t*-test. The correlation between the two groups of samples was evaluated using the Pearson correlation coefficient. The correlation between the ERRα expression and pathological indexes of the patients was calculated using Spearman’s correlation analysis. The Kaplan–Meier method was applied to analyse patient survival, and the log-rank test was adopted to estimate differences between groups. Overall, survival was defined as the interval from the date of initial surgery to the date of death or last contact. Patients who lost contact were treated as survivors. The Cox proportional hazards regression model was used for multivariate analysis to study the contribution of various potential prognostic parameters to OS.

All the data were presented as mean ± standard error. Significance was established as *P*<0.05. All the analyses were performed using SPSS 22.0 software.

### Ethics approval and consent to participate

The study was approved by the Ethics Committee of the Department of thoracic surgery of Jiangsu Cancer Hospital and written informed consent was obtained from each participant.

### Availability of data and materials

All of the data supporting our findings can be found in the main paper. All data of the data generated in this study are available upon request by writing to the corresponding author.
